# Supplementary material for: Splice-Junction-Based Mapping of Alternative Isoforms in the Human Proteome
Source: Cell Rep. Author manuscript; Available in PMC 2020 Jan 15. (PMC6961840; doi:10.1016/j.celrep.2019.11.026)
Supplement: 2 [file NIHMS1546469-supplement-2.zip › DF1/PXD006675/RightVentricle/RightVentricle_50_MAPKAPK5_QVIEEQTTSHESTGGAK.pdf]

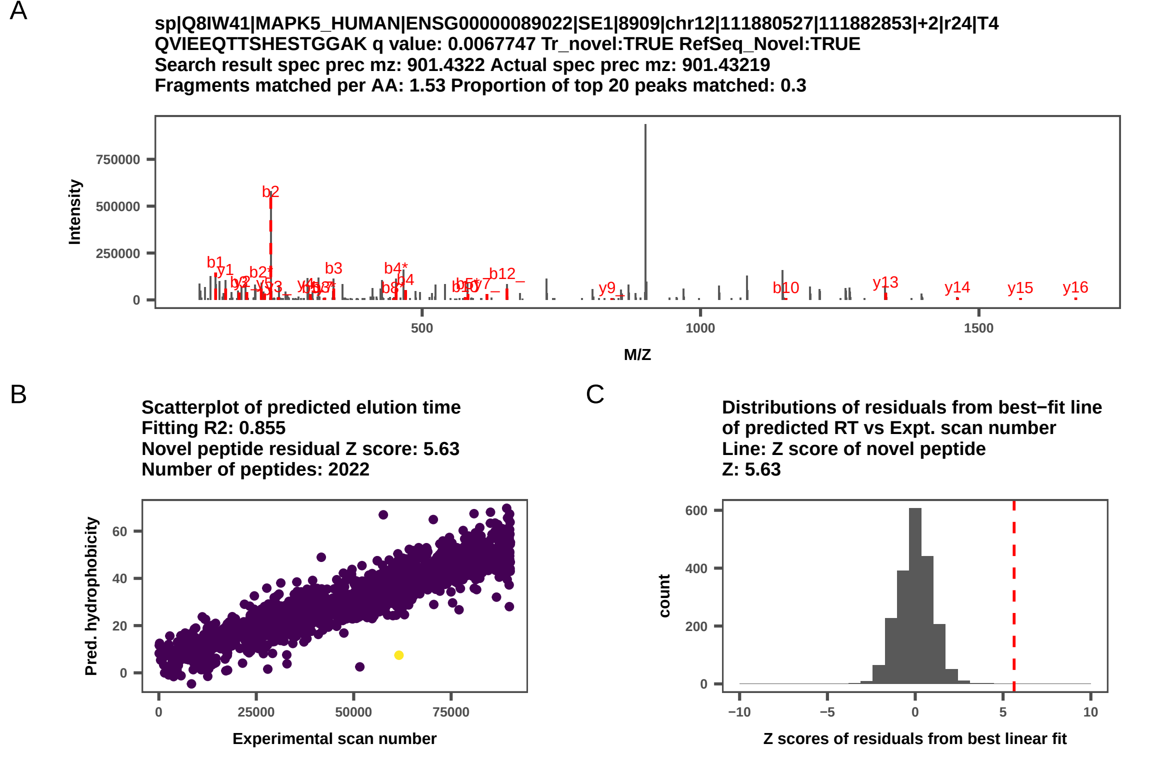
A
sp|Q8IW41|MAPK5_HUMAN|ENSG00000089022|SE1|8909|chr12|111880527|111882853|+2|r24|T4
QVIEEQTTSHESTGGAK q value: 0.0067747 Tr_novel:TRUE RefSeq_Novel:TRUE
Search result spec prec mz: 901.4322 Actual spec prec mz: 901.43219
Fragments matched per AA: 1.53 Proportion of top 20 peaks matched: 0.3
Intensity
750000
500000
250000
0
b2
b1
y1
b2*
b3
b4*
y4
b8*
b12
y9
b10
y13
y14
y15
y16
500
1000
1500
M/Z
B
Scatterplot of predicted elution time
Fitting R2: 0.855
Novel peptide residual Z score: 5.63
Number of peptides: 2022
Pred. hydrophobicity
60
40
20
0
25000
50000
75000
Experimental scan number
C
Distributions of residuals from best−fit line
of predicted RT vs Expt. scan number
Line: Z score of novel peptide
Z: 5.63
count
600
400
200
−10
−5
5
10
Z scores of residuals from best linear fit
